# High genetic relatedness between multidrug resistant bacteria before and after the 2022 invasion of Ukraine

**DOI:** 10.1186/s13073-025-01500-1

**Published:** 2025-07-01

**Authors:** Francois Lebreton, Viacheslav Kondratiuk, Valentyn Kovalchuk, Niels Pfennigwerth, Ting L. Luo, Brendan T. Jones, Nadiia Fomina, Frieder Fuchs, Jörg B. Hans, Jessica Eisfeld, Ana Ong, Sören Gatermann, Jason W. Bennett, Patrick Mc Gann

**Affiliations:** 1 Multidrug-Resistant Organism Repository and Surveillance Network (MRSN), Diagnostics and Countermeasures Branch, CIDR, Walter Reed Army Institute of Research, 503 Robert Grant Ave, Room 2A36, Silver Spring, MD 20910 USA; 2 Department of Emergency and Military Medicine, National Pirogov Memorial Medical University, Vinnytsia, Ukraine; 3 Department of Microbiology, National Pirogov Memorial Medical University, Vinnytsia, Ukraine; 4 National Reference Centre for Multidrug-Resistant Gram-Negative Bacteria, Department of Medical Microbiology, Ruhr-University Bochum, Bochum, 44801 Germany; 5 Department of Microbiology and Hospital Hygiene, Bundeswehr Central Hospital Koblenz, Koblenz, 56072 Germany; 6 Landesuntersuchungsamt Rheinland-Pfalz, Koblenz, 56068 Germany; 7 Institute for Medical Microbiology, Immunology and Hygiene, Medical Faculty, University of Cologne, University Hospital of Cologne, Cologne, 50931 Germany

**Keywords:** *A. baumannii*, *P. aeruginosa*, Surveillance, Carbapenem resistance, Nosocomial outbreak, Microbial genomics, Antimicrobial resistance, Molecular epidemiology

## Abstract

**Background:**

The Russian invasion of Ukraine in 2022 has placed extraordinary pressure on hospitals there. One consequence of this has been the alarming increase in infections caused by multi-drug resistant organisms (MDROs), both within Ukraine and among the Ukrainian diaspora. The original source of these MDROs remains obscure although nosocomial origin is suspected. Here, we analyzed a collection of *Acinetobacter baumannii* and *Pseudomonas aeruginosa* collected from Ukraine before and after the invasion to glean a greater understanding of their relationship and origins.

**Methods:**

Genomic analysis was conducted on 167 *A. baumannii* and 93 *P. aeruginosa* cultured from 223 Ukrainian patients hospitalized in Ukraine or other European countries. Fifty-three isolates were cultured between 2014 and 2021, prior to the invasion, and the remaining 207 after.

**Results:**

Highly genetically related extensively-drug resistant (XDR) clones were identified that spanned the pre- and post-invasion periods. For *A. baumannii*, isolates encompassed three sequence types (STs), including carbapenemase-producing strains from ST-2 (*bla*_OXA-23_) and ST-78 (*bla*_OXA-72_), as well as ST-400 carrying the ESBL *bla*_GES-11_. For *P. aeruginosa*, isolates encompassed three STs: ST-773 carrying *bla*_NDM-1_, ST-1047 carrying *bla*_IMP-1_, and ST-244. For all, the mobile genetic elements associated with carbapenemase carriage were fully characterized. Notably, post-invasion ST-773 and ST-1047 *P. aeruginosa* had a signature of host adaptation with multiple loss-of-function mutations in the quorum-sensing regulator LasR, known to modulate immune responses and provide survival advantages in animal models of infection.

**Conclusions:**

XDR epidemic clones circulating in Ukraine and across Europe since 2022 share a close genetic relationship to historical strains from Ukraine. In some cases, direct links to medical facilities within Ukraine can be inferred. These data suggest that surveillance efforts should focus on tracking nosocomial transmission within Ukrainian hospitals while infection control efforts are being disrupted by the ongoing Russian invasion.

**Supplementary Information:**

The online version contains supplementary material available at 10.1186/s13073-025-01500-1.

## Background

Human conflict plays an important role in the development and spread of antimicrobial resistance (AMR) [1], and the current war in Ukraine is no exception [2]. The Russian invasion has placed extraordinary pressure on Ukrainian healthcare services, with medical facilities struggling with large numbers of casualties while dealing with constant bombing, constrained supply chains, and greatly reduced ability to provide basic services [3]. One area where these challenges have been acutely felt is infection control, where large increases in multi-drug resistant (MDR) and extensively-drug resistant (XDR) bacteria have been reported among wounded service members and civilians receiving care in Ukrainian hospitals [4–6].

Prior to the invasion in February 2022, Ukraine was engaged in a conflict with separatists in the Donbas and Luhansk regions of Eastern Ukraine since 2014. Bacterial infections among injured service members during that Eastern conflict were characterized by initial contamination with Gram-positive microbes of low pathogenicity that gradually transitioned to more pathogenic Gram-negative rods during the wound healing process [7]. Notably, non-fermentative Gram-negative bacilli accounted for 68% of all positive wound swab cultures after the first week of healing, with *Acinetobacter baumannii* and *Pseudomonas aeruginosa* being the most prevalent [7].

A genetic analysis of these earlier (2014–2020) isolates from Ukraine revealed *A. baumannii* belonging to sequence type (ST)−1^PAS^ and ST-78^PAS^ and *P. aeruginosa* belonging to ST-235 and ST-773 carrying an array of AMR determinants, including the class D carbapenemases OXA-23 and OXA-72 in *A. baumannii* and the class B carbapenemase VIM-2 in *P. aeruginosa* [8]. These strains also carried 16S methyltransferases genes (16S RMTases; *armA* and *rmtB4*, respectively), and ST-78^PAS^ *A. baumannii* also carried the extended spectrum β-lactamase (ESBL) genes *bla*_CTX-M-115_. Notably, since 2022, MDR and XDR strains from these lineages have been cultured from Ukrainian refugees and military personnel receiving medical care in other European countries [9–13], suggestive of a common reservoir before evacuation.

The origins of these MDR and XDR strains remains obscure and while historical isolates have been described at the lineage level [8], no comprehensive genomic comparison has been made between pre-invasion and post-invasion isolates. Here, we conduct such analysis on *A. baumannii* and *P. aeruginosa* and show that ongoing outbreaks are largely due to MDR and XDR clones already circulating in Ukraine before the invasion, and whose spread is now likely exacerbated because of the war.

## Methods

### Strain collection

Two hundred and sixty genomes, representing 260 isolates cultured from 223 patients, were available for analysis. Fifty-three were collected between 2014 and 2021 (pre-invasion) and 207 between March 2022 and September 2023 (post-invasion) (Table S1, [14]). Of these 260 isolates, 173 isolates from 136 patients were physically available for additional testing. These 173 isolates consisted of 161 cultured at eight Ukrainian medical facilities and 12 cultured at two German medical facilities (Table S1, [14]). All 161 Ukrainian isolates (45 pre-invasion and 116 post-invasion) were cultured from injured Ukrainian service members with clinical evidence of infection (edema, erythema, lymphangitis, wound discharge/purulence). The majority (63%) were recovered from wound cultures, followed by surveillance swabs (14.6%, rectal and groin), respiratory (8.5%), burn (4.2%), blood (3.8%), or various other (5.8%) cultures. Isolates were cultured from patients using two nutrient media: tryptone soy agar and chromogenic agar for *Acinetobacter* (Graso Biotech, Poland). For post-invasion isolates, the soldiers had been injured 6.2 ± 3.9 days prior to culture and had been transferred through 4–5 evacuation hospitals ranging from NATO level II (qualified medical care) to III (specialized medical care) [15] before arriving at the final facility. All 12 German isolates (1 pre-invasion and 11 post-invasion) were cultured from soldiers injured in Ukraine and receiving care at two military hospitals in Germany. Notably, the single pre-invasion German isolate (MRSN 483036) was cultured in 2017 from a peri-rectal surveillance swab of a US Army servicemember transferred to Landstuhl Regional Medical Center (LRMC) in Germany after prior hospitalization in Ukraine following a motor vehicle collision there.

These 173 isolates were supplemented with 87 genomes of isolates collected as part of surveillance programs in Denmark (*n* = 3), Germany (*n* = 75), and The Netherlands (*n* = 9). Sixty-six of the genomes from Germany were provided by the National Reference Centre for Multidrug-Resistant Gram-Negative Bacteria at Ruhr-University Bochum, Germany and were collected as part of a nationwide surveillance program. The remaining nine genomes were obtained from the National Center for Biotechnology Information (NCBI) and represent five and four isolates cultured from Ukrainian patients receiving care at German facility #3 and Germany facility #5, respectively (no associated publications). Similarly, the Danish and Dutch genomes were obtained from NCBI and represent bacteria collected from Ukrainian patients receiving care at a medical facility in The Netherlands and Denmark (no associated publication).

### Antibiotic susceptibility testing (AST)

AST was performed on all 173 isolates using a Vitek 2 (Biomerieux) with cards N808 and XN-32, or broth microdilution, as previously described [16]. Strains were defined as MDR if they were resistant to ≥ 1 agent in three categories and XDR if they were non-susceptible to ≥ 1 agent in all but ≤ 2 categories of the tested antimicrobials.

### DNA extraction, whole genome sequencing (WGS), and de novo assemblies

- Short-read sequencing:
  DNA was extracted using the DNeasy UltraClean 96 Microbial Kit (Qiagen, Germantown, MD, USA) and libraries were constructed using the KAPA Hyperplus Library preparation kit (Roche Diagnostics, Indianapolis, IN, USA). Libraries were quantified using the KAPA Library Quantification Kit—Illumina/Bio-Rad iCycler™ (Roche Diagnostics) on a CFX96 real-time cycler (Biorad, Hercules, CA, USA). Libraries were normalized to 2 nM, pooled, denatured, and diluted to 1 nM. Whole genome sequencing was performed using a MiSeq, NextSeq 500, or NextSeq 2000 Benchtop Sequencer (Illumina Inc., CA, USA) with MiSeq Reagent Kit v3 (600 cycles; 2 × 300 bp), NextSeq Reagent Kit 500/550 v2 (300 cycles; 2 × 150 bp), or NextSeq 1000/2000 P2 Reagents (300 cycles) v3 kit (Illumina, San Diego, CA, USA). bbduk v38.96 [17] was used to remove barcode and adapter sequence as well as to perform quality trimming (ktrim = “*r*”, k = “23”, mink = “11”, hdist = “1”, qtrim = “*r*”, trimq = “15”, minlen = “100”). Kraken2 v2.1.2 [18] was used for initial taxonomic assignment (top hit = “1”, undetermined reads = “ < 10%”) and to screen for contamination (2 + genus level hits > 5% = “isContaminated”). De novo draft genome assemblies were produced using shovill v1.1.0 [19] with coverage estimates generated using bbmap v38.96 [20]. Minimum thresholds for contig size and coverage were set at 200 bp and 49.5 + , respectively. Quality controls for the assembly were standardized with a decision tree including the following parameters: total length of contigs > 1 Mb; total length of contigs < 1 Mb over expected genome size for taxon; average read depth for each contig ≥ 20; total length of contigs filtered for low coverage < 100 kb; total length of contigs filtered for length < 100 kb; and number of contigs filtered for length + numbers of contigs filtered for low coverage < 1000. In cases where the Kraken2-derived taxonomic assignment was ambiguous, GTDB [21] was used via the GTDB-Tk v2.4.0 [22] and a > 95% average nucleotide identity threshold for species level identification.
- Long-read sequencing:
  Long-read sequencing was performed on a Minion platform using a MinION Mk1B device (Oxford Nanopore Technologies, Oxford, England). DNA was extracted using the DNeasy UltraClean Microbial Kit (Qiagen, Germantown, MD, USA). Library preparation on genomic DNA was performed using SQK-RBK114-96 rapid barcoding kit and sequenced on a 10.4.1 flowcell. The resulting POD5 output were basecalled with Dorado V9.0.0 [23] using the super-accurate model dna_r10.4.1_e8.2_400bps_sup@v5.0.0. After demultiplexing fastq into samples, a hybrid long-read/short-read assembly approach was used to assemble genomes. First, de novo assembly from long reads only was performed using Autocycler v0.1.2 [24]. Briefly, Autocycler outputs a consensus assembly from multiple long-read dedicated assemblers. The dedicated assemblers used were Flye, Raven, Miniasm, Metamdbg, Necat, NextDenovo, and Redbean. Next, the consensus assembly was polished with Illumina short reads using Polypolish [25].

### Molecular typing and annotations

Genomes were annotated using Bakta v.1.10.4 [26]. Antimicrobial resistance genes were annotated using a combination of ARIBA v2.14.6 [27] and AMRFinderPlus v3.12.8 [28] and the following parameters: ident_min < 0.9 > and coverage_min < 0.5 > for AMRFinderPlus; nucmer_min_id < 90 > nucmer_min_len < 20 > and nucmer_breaklen < 200 > for ARIBA. Finally, a 10 × minimum coverage was applied for ARIBA-only hits and deduplication was performed with priority given to the assembly-based hit. MLST assignment was performed using mlst v2.22.1 [29]. Mobile genetic elements were identified and annotated with tools and databases such as mobileOG-db (beatrix-1.6) [30], ISFinder [31], and TnCentral [32]. Plasmid replication initiation genes (Rep types) were determined using the Acinetobacter Plasmid Typing scheme [33] and plasmidFInder [34]. Comparisons of mobile genetic elements (MGE) were generated using the BLAST comparison tool version 1.4.1 integrated in Proksee.ca [35]. Parameters included an expect value cutoff of 0.0001, filtering of low complexity regions, and filtering of regions with < 95% nucleotide identity. When necessary, manual annotation and superficial edits (e.g., adding a sequence break on Fig. 6A) were made for the final illustration in CorelDRAW.

### Core genome MLST, SNP calling, and phylogenetic analysis

Detection of clusters of high genetic relatedness was performed in two stages. First, core genome MLST was performed in SeqSphere + Software v 7.7.2 (Ridom, Germany) using the cgMLST schemes developed for *P. aeruginosa* [36] and *A. baumannii* [37] with a cutoff of 90%. Isolates related by ≤ 20 alleles were considered part of a cluster of interest and additional whole genome single nucleotide polymorphism (SNP) analysis was performed. While more stringent thresholds (e.g., ≤ 5 allelic differences) have been applied to identify transmission clusters within a single hospital or healthcare network [38], we chose this conservative threshold in agreement with other studies of prolonged outbreaks [39, 40]. This was done to better suit our dataset which spanned 4 countries for up to 8 years and was not the result of a systematic and thorough sampling/surveillance strategy. As a result, we did not aim to reconstruct local patient-to-patient transmission but rather to track the spread of epidemic clones with origins from Ukraine. The cgMLST minimum-spanning tree was generated using the species-specific cgMLST schemes and allelic distance matrices. When necessary, superficial edits (e.g., changing node shape from circle to square) were made in CorelDRAW for the final illustration.

Second, to further investigate these putative clusters, an internal reference genome (first isolate temporally) was picked and whole genome SNP analysis was individually performed. SNP calling was performed with Snippy v.4.4.5 [41] using error corrected (Pilon v1.23) [42] and annotated draft assemblies with the earliest isolate (by date) within each cgMLST cluster chosen as reference. For each cluster, the core SNP alignment (with respective lengths of 3,972,412 bp for *A. baumannii* ST-2; 4,074,973 bp for *A. baumannii* ST-78 cluster 1; 4,123,489 bp for *A. baumannii* ST-78 cluster 2; 4,134,951 bp for *A. baumannii* ST-78 cluster 3; 4,022,063 bp for *A. baumannii* ST-400; 6,850,243 bp for *P. aeruginosa* ST-773; and 7,070,241 bp for *P. aeruginosa* ST-1047) was filtered for recombination using Gubbins v2.3.156 [43] and a SNP-based phylogeny was created by inferring a maximum-likelihood tree with RaxML-NG v0.9.0 [44] using the GTR + G model and 50–50 parsimony and random starting trees.

## Results

### Identification of clusters of high genetic relatedness grouping pre- and post-invasion isolates

**Fig. 1:**
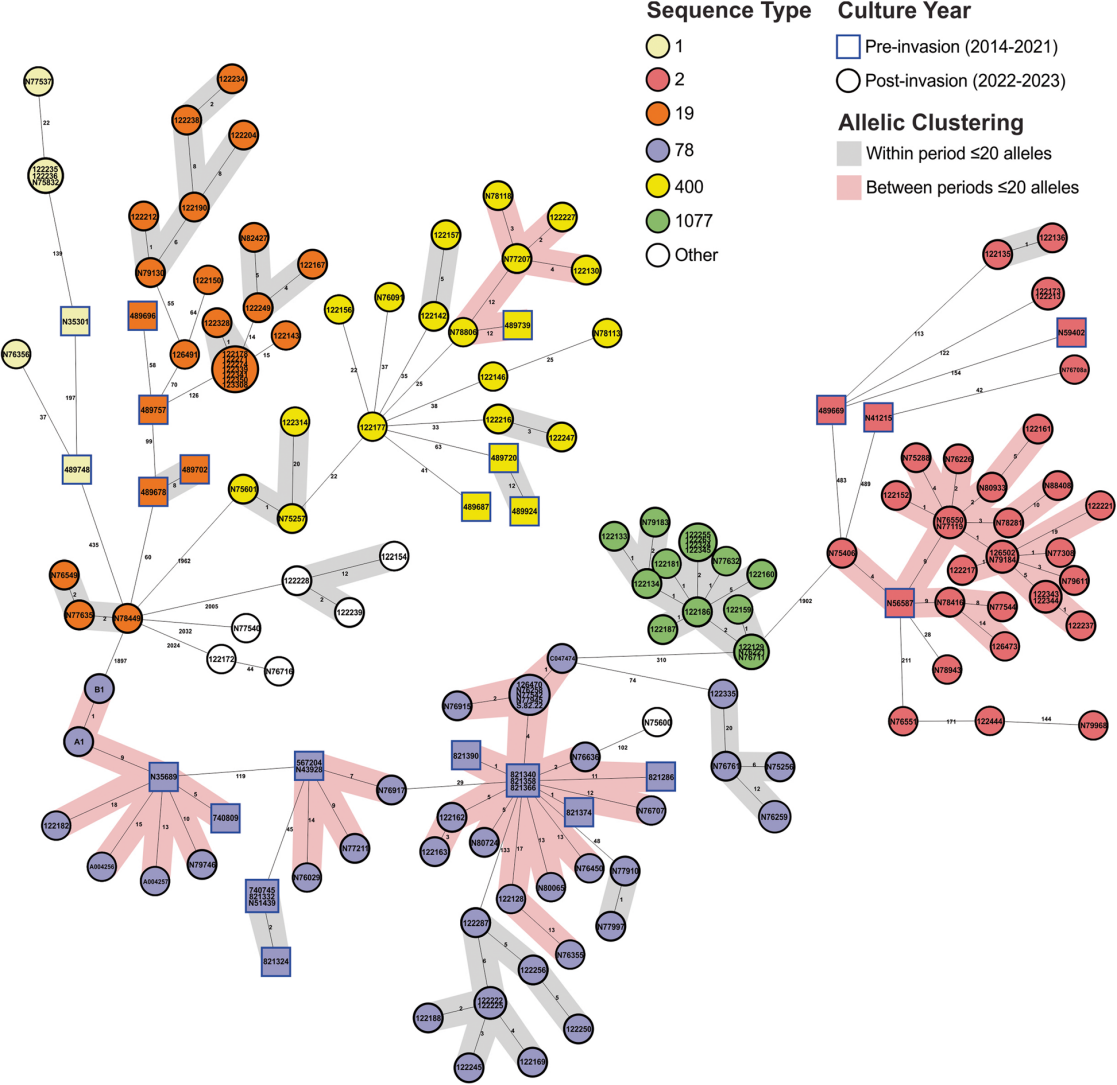
Genetic relationship of all *A. baumannii* in this study. Minimum spanning tree of all 167 *A. baumannii* analyzed in this study. Isolates are colored by sequence type and numbers between nodes are core genome MLST allelic differences and are highlighted if ≤ 20 alleles. Isolate nodes are shaped based on year of collection with square nodes indicating isolates collected pre-invasion and circular nodes indicating isolates collected post-invasion

**Fig. 2:**
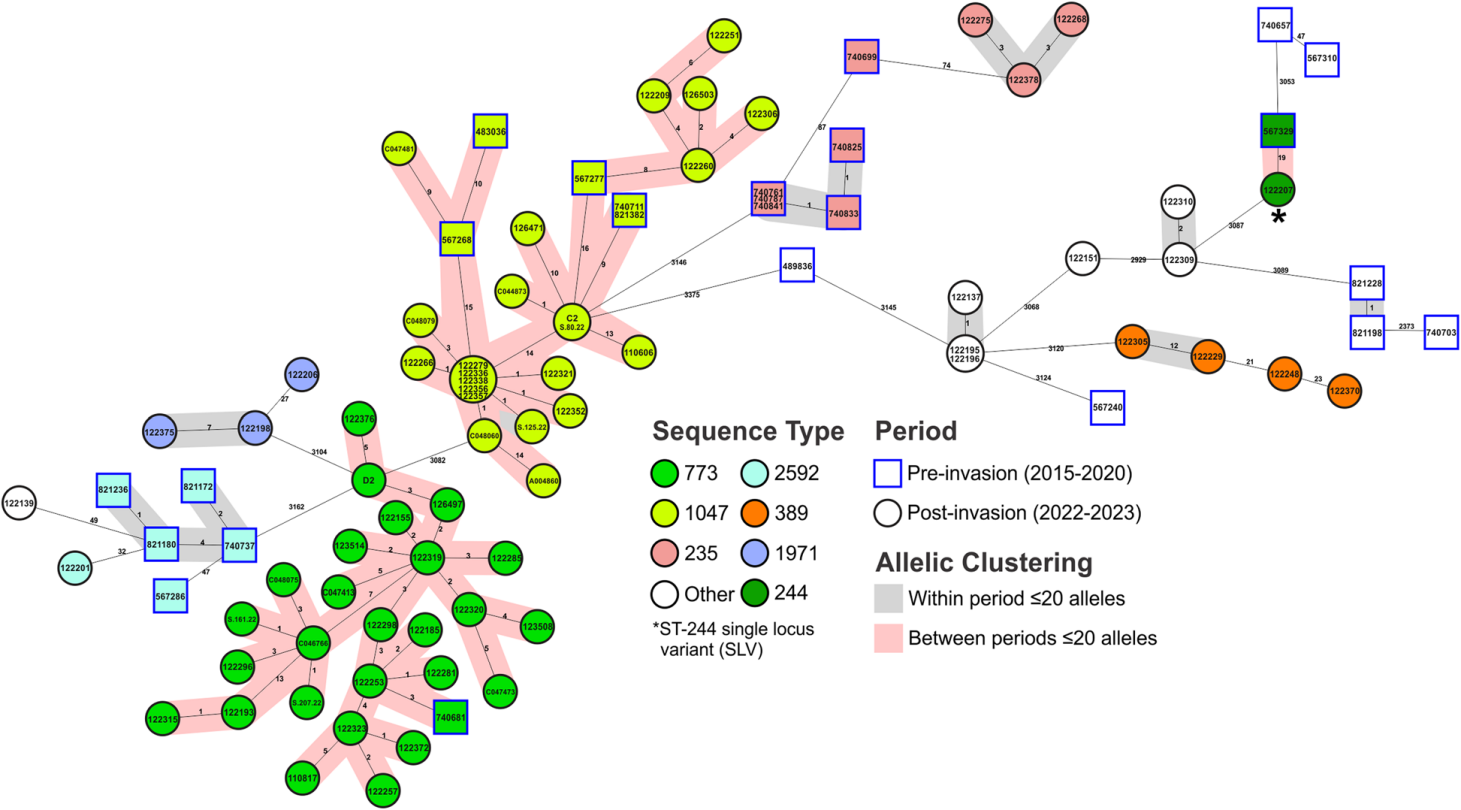
Genetic relationship of all *P. aeruginosa* isolates in this study. Minimum spanning tree of all 93 *P. aeruginosa* analyzed in this study. Isolates are colored by sequence type and numbers between nodes are core genome MLST allelic differences and are highlighted if ≤ 20 alleles. Isolate nodes are shaped based on year of collection with square nodes indicating isolates collected pre-invasion and circular nodes indicating isolates collected post-invasion

Of the 260 available genomes, 167 were *A. baumannii* and 93 were *P. aeruginosa* (Table S1, [14]). The *A. baumannii* were distributed across 10 different ST, with 28 isolates collected pre-invasion and 139 collected post-invasion. Notable globally distributed and clinically important clones were identified, including ST-2 with the *bla*_OXA-23_ or *bla*_OXA-72_ carbapenemase genes, ST-78 with *bla*_OXA-72_, and ST-400 with the ESBL gene *bla*_GES-11_ (Fig. 1). *P. aeruginosa* were distributed across 17 STs, with 25 collected pre-invasion and 68 post-invasion. High-risk clones were also identified, including ST-773 and ST-1047 carrying the *bla*_NDM-1_ and *bla*_IMP-1_ carbapenemase genes, respectively (Fig. 2).

Though distinct ST clusters were identified pre- and post-invasion, five *A. baumannii* and five *P. aeruginosa* STs were present during both periods (Figs. 1 and 2). Two of the *A. baumannii* STs (1 and 19) and two of the *P. aeruginosa* STs (235 and 2592) had pre-invasion isolates that were separated from the post-invasion isolates by > 20 allelic differences, and no further analysis was performed. The remaining three *A. baumannii* (ST-2, −78, and −400) and *P. aeruginosa* STs (ST-244, −773, and −1047) contained pre- and post-invasion isolates that were separated by < 20 allelic differences and these were selected for further SNP-based analysis (Table S2, [14]). This revealed that pre- and post-invasion strains differed by 3–87 SNPs across all isolates, though significant variation was observed between the six clusters.

### An endemic clone of *A. baumannii* ST-2 in Ukraine and Germany

**Fig. 3:**
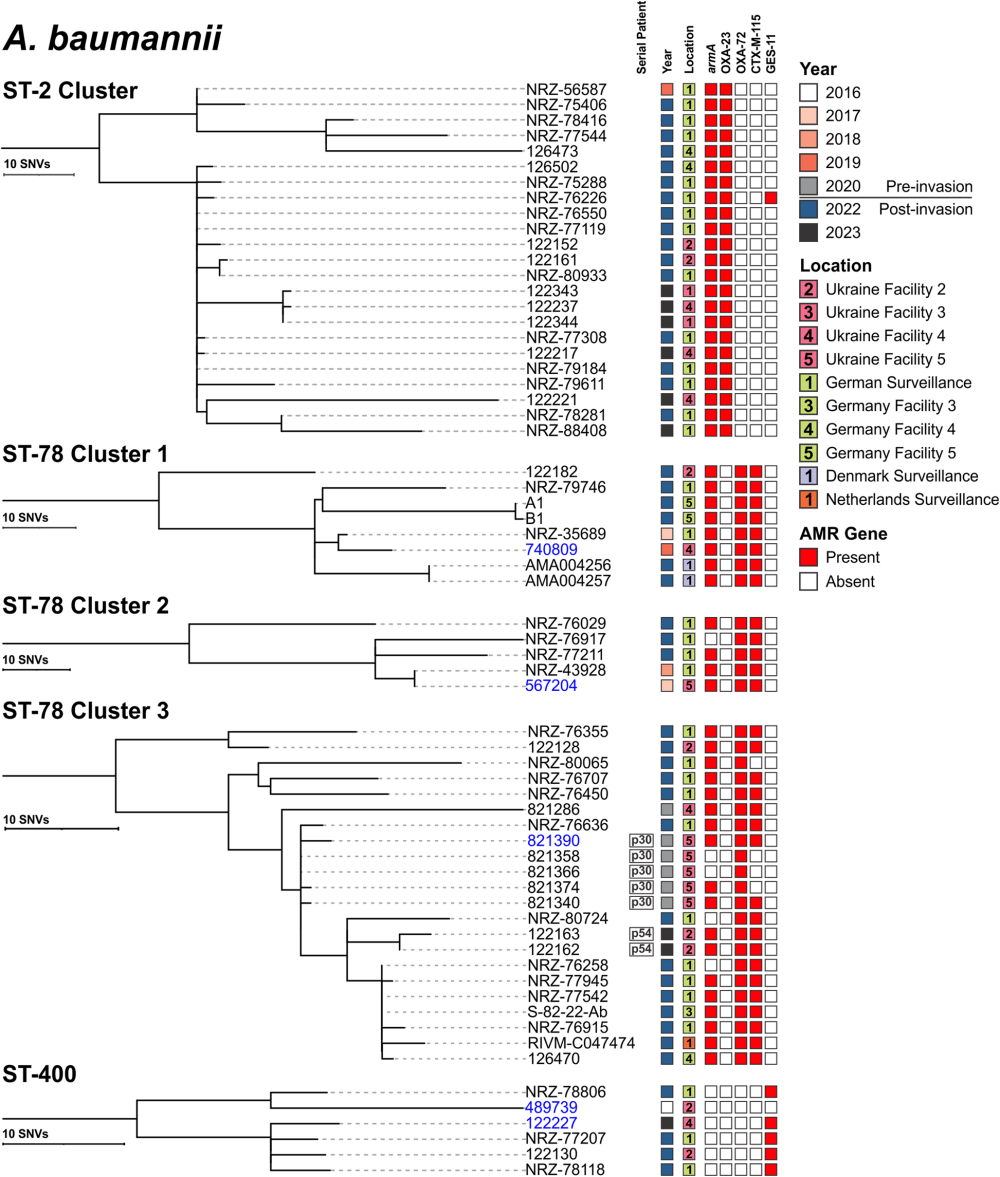
Single nucleotide variant phylogenies of select *A. baumannii* cgMLST clusters from three lineages. Isolate metadata include year and facility of culture collection, and AMR genes detected. Isolates sequenced with long-read platform(s) are shown in blue. All presented epidemic clones were detected in both European and Ukrainian facilities and included pre-/post-invasion isolates

**Fig. 4:**
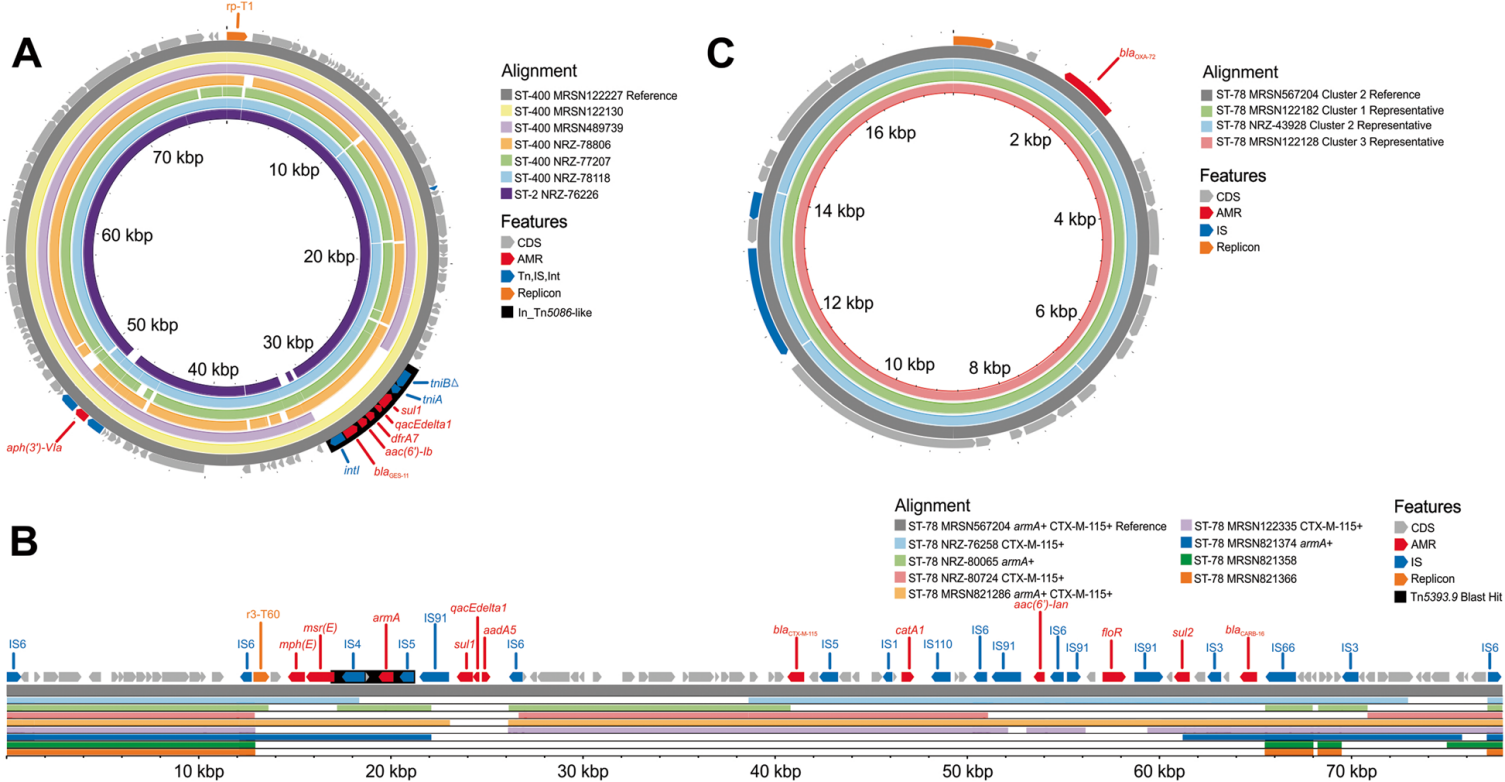
Genetic context of major resistance genes in *A. baumannii* epidemic clones. **A** Plasmid-encoded *bla*_GES-11_ in ST-2 and ST-400 isolates. Reference is circularized 77.6 kb plasmid from ST-400 MRSN122227. Short-read contig mapping to the reference revealed that isolates with *bla*_GES-11_ possessed the Tn*5086*-like transposon, including ST-2 isolate NRZ-76226. **B** Chromosomal co-localization of *armA* and *bla*_CTX-M-115 in_ ST-78 isolates. Reference is a 77.7 kb chromosomal fragment from MRSN567204. Short-read contig mapping revealed that the region is highly recombinant. **C** Plasmidic *bla*_OXA-72_ in ST-78 isolates. Reference is circularized 17.8 kb plasmid from MRSN567204

Although 35 isolates were assigned to ST-2, only 23 (16 from multiple hospitals in Germany and seven from 2 hospitals in Ukraine) formed a distinct cluster that contained both pre- and post-invasion isolates separated by ≤ 20 allelic differences (Figs. 1 and 3). All isolates carried *armA* and *bla*_OXA-23_. Only NRZ-56587, cultured from a Ukrainian patient being treated in a German hospital, was cultured before the invasion (2019), with the remaining isolates being collected in 2022 (*n* = 16) or 2023 (*n* = 6) (Fig. 3). Notably, NRZ-56587 was genetically nearly identical (5 SNPs) to NRZ-75406 collected during national surveillance 3 years later and shared a high level of genetic relatedness (16–54 SNPs) with the other 21 contemporary isolates from both Ukraine and Germany. A subset of 16 contemporary (2022–2023) isolates from German and Ukrainian hospitals were distinct by only 0–17 SNPs (Fig. 3). Interestingly, one isolate (NRZ-76226) acquired the ESBL gene *bla*_GES-11_, inserted as the first cassette within an Tn*5086*-like transposon, itself carried by a putative plasmid sharing homologies (99.99% nucleotide identity and 91.27% coverage) with a 77 kb rp-T1 type plasmid found in the six ST-400 *A. baumannii* collected from facilities in both Ukraine and Germany (Fig. 4A).

### Three distinct clones of *A. baumannii* ST-78 detected in Ukraine and other European countries

A high degree of genetic diversity was observed within ST-78, and cgMLST segregated the 53 isolates into 5 broad clusters that were separated by up to 133 allelic variants (Fig. 1). Of these, 35 isolates formed 3 clusters with ≤ 20 allelic differences that contained both pre- and post-invasion isolates (Fig. 1, Table S2). Ten isolates (from 6 patients) were collected pre-invasion, eight from 4 Ukrainian soldiers injured during the Eastern conflict in 2017, 2019, and 2020, and two from Ukrainian soldiers receiving treatment in Germany in 2017 and 2018 (Table S2, [14]). The remaining 25 isolates were cultured post-invasion from injured Ukrainian civilians and military receiving care in Denmark (*n* = 2), Germany (*n* = 18), The Netherlands (*n* = 1), and Ukraine (*n* = 4). Twenty-eight of the 35 isolates carried *bla*_OXA-72_, the ESBL gene *bla*_CTX-M-115_, and *armA* (Fig. 3). Long-read sequencing revealed that *armA* and *bla*_CTX-M-115_ were chromosomally integrated on a large 77.7 kb fragment (in reference genome MRSN567204), flanked by insertion sequence (IS) 26-like, part of the IS*6* family (Fig. 4B). Similar islands, detected by Vuillemenot et al. in *bla*_CTX-M-115_-carrying *A. baumannii* from France, ranged from 20 to 79 kb in size and contained different combinations of determinants encoding multiple resistances and flanked by several ISs promoting possible sequence rearrangements [45]. Here, with partial loss of internal sections of the resistance island, five isolates lacked *armA* while *bla*_CTX-M-115_ was missing in four isolates (Fig. 4B). Finally, in every isolate, *bla*_OXA-72_ was encoded on a 15–17 kb plasmid (Fig. 4C) with a replication origin identical to that of pMMA2 [46], and high homologies (> 99.9% nucleotide identity) with pAbCTX5 from a ST-78 isolate from France [45].

SNP-based analysis of the three sub-clusters revealed that within each sub-cluster, isolates differed by 0–55 SNPs; with pre-invasion isolates differing from post-invasion isolates by 2–55 SNPs (Fig. 3).

Sub-cluster 1 encompassed 8 isolates from three countries (Ukraine (*n* = 2), Germany (*n* = 4), and Denmark (*n* = 2)), with two (NRZ-35689 and 740,809) being collected before the full-scale invasion (2017 and 2019 from Germany and Ukraine, respectively). Both pre-invasion isolates differed from one another by 7 SNPs and were separated from post-invasion isolates by 12–38 SNPs (Fig. 3). Two isolates cultured from Ukrainian patients receiving care in Denmark in 2022 were separated from the German isolate NRZ-35689 by just 12–14 SNPs, despite having been cultured over 5 years apart.

Sub-cluster 2 encompassed five isolates from two countries (Ukraine (*n* = 1) and Germany (*n* = 4)) (Fig. 3). Two isolates were collected pre-invasion: one from Ukraine in 2017 (567,204) and the other from a Ukrainian soldier receiving care in a German hospital in 2018 (NRZ-43928). Notably, both pre-invasion isolates were genetically identical (0 SNPs), suggesting that both patients were exposed to the same reservoir, possibly in Ukraine facility #5. Unfortunately, no further patient movement information is available to test this hypothesis further. Finally, sub-cluster 3 encompassed 22 isolates from three countries: Germany (*n* = 12), Ukraine (*n* = 9), and The Netherlands (*n* = 1) (Fig. 2). Six isolates were collected pre-invasion including five highly genetically related isolates cultured from the same patient (P30) in Ukraine in May 2020 (Table S1 and Fig. 3). Though all isolates were separated by up to 55 SNPs, the 5 pre-invasion isolates from patient P30 were separated from NRZ-76636 by just 2–5 SNPs. This latter isolate was obtained as part of the national German surveillance program from an injured Ukrainian soldier in May 2022, but no further patient information is available.

### Acquisition of an ESBL in an epidemic clone of *A. baumannii* ST-400

Of 21 isolates assigned to ST-400, just six (three each from Germany and Ukraine) met the criteria for inclusion (Fig. 1). Only MRSN 489739 was cultured prior to the invasion, from an injured Ukrainian soldier in 2016 at Ukraine facility #2 (Fig. 3). Though at least 6 years separates 489,739 from the other isolates, they differ by just 15–30 SNPs, with the closest relative being NRZ-78806, cultured in August 2022 from a wound of an injured Ukrainian soldier being treated in a German hospital. Notably, though none of the isolates carries an acquired carbapenemase, all five post-invasion isolates acquired *bla*_GES-11_ (within a Tn*5086*-like transposon harbored by an ~ 70 kb rp-T1 type plasmid, as discussed above, Fig. 4A), a variant of the ESBL *bla*_GES-1_ that confers increased carbapenem resistance [47]. In accordance, the 5 contemporary isolates were non-susceptible to ampicillin-sulbactam (≥ 32/16 µg/ml), ceftazidime (≥ 32 µg/ml), meropenem (≥ 38 µg/ml), and trimethoprim-sulfamethoxazole (≥ 4/76 µg/ml) while 489,739 (historical) remained susceptible.

### Endemic NDM-carrying *P. aeruginosa* ST-773 clone shows signature of host adaption as it spreads from Ukraine

**Fig. 5:**
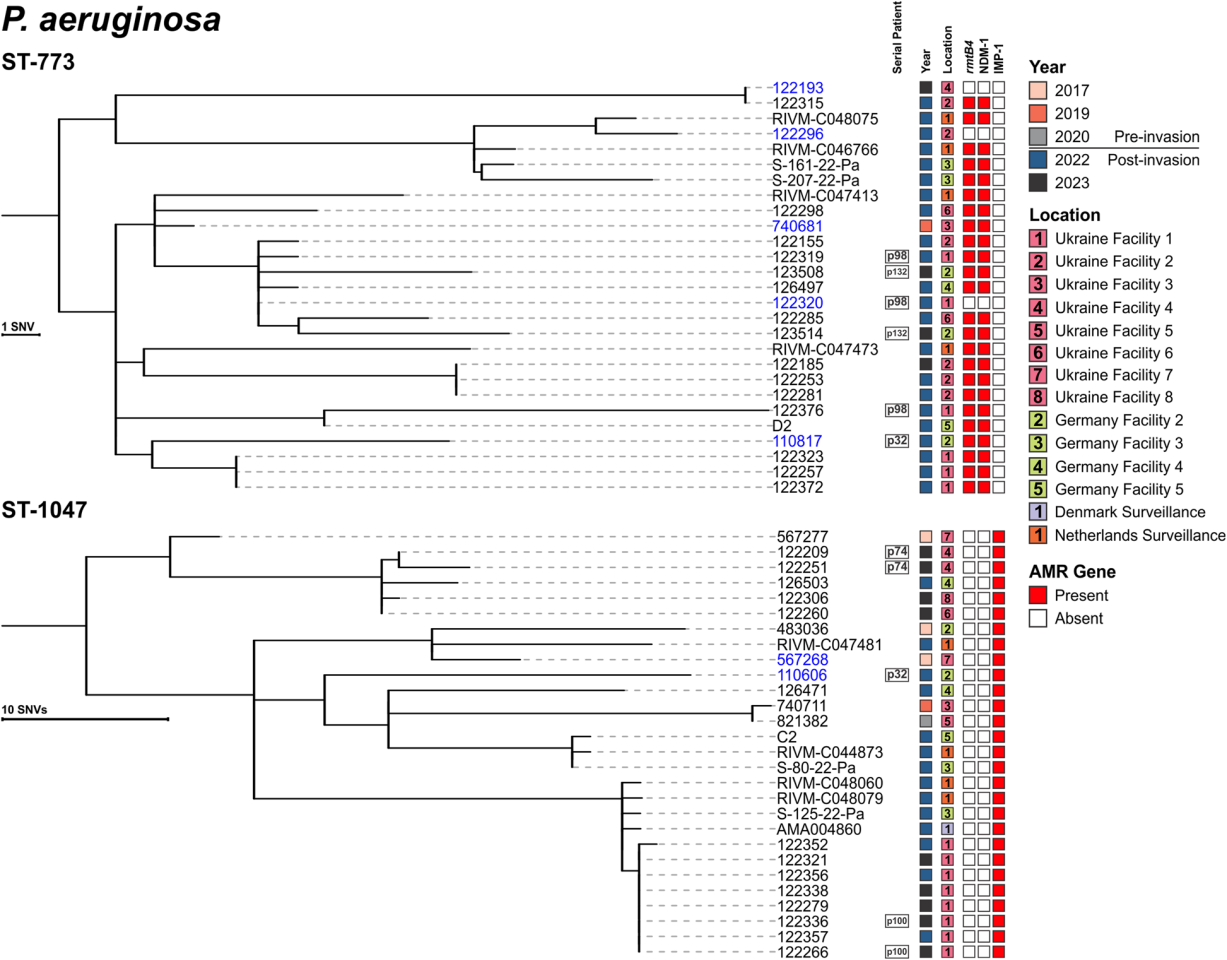
Single nucleotide variant phylogenies of two *P. aeruginosa* cgMLST clusters from two lineages. Isolate metadata include year and facility of culture collection, and AMR genes detected. Isolates collected from the same patient are indicated by the serial patient, if applicable. Isolates sequenced with long-read platform(s) are shown in blue

Twenty-seven isolates from three countries (Ukraine (*n* = 16), Germany (*n* = 7), and The Netherlands (*n* = 4)) were assigned to ST-773 (Fig. 2). All formed a cluster of highly related isolates, with just MRSN 740681 being cultured before the invasion (2019) at Ukraine facility #3 (Fig. 5). SNP-based analysis indicated that the isolates were separated by 0–32 SNPs and the 2019 isolate was separated from all other isolates by just by 4–18 SNPs, with 8 recent Ukrainian isolates being separated by ≤ 5 SNPs.

**Fig. 6:**
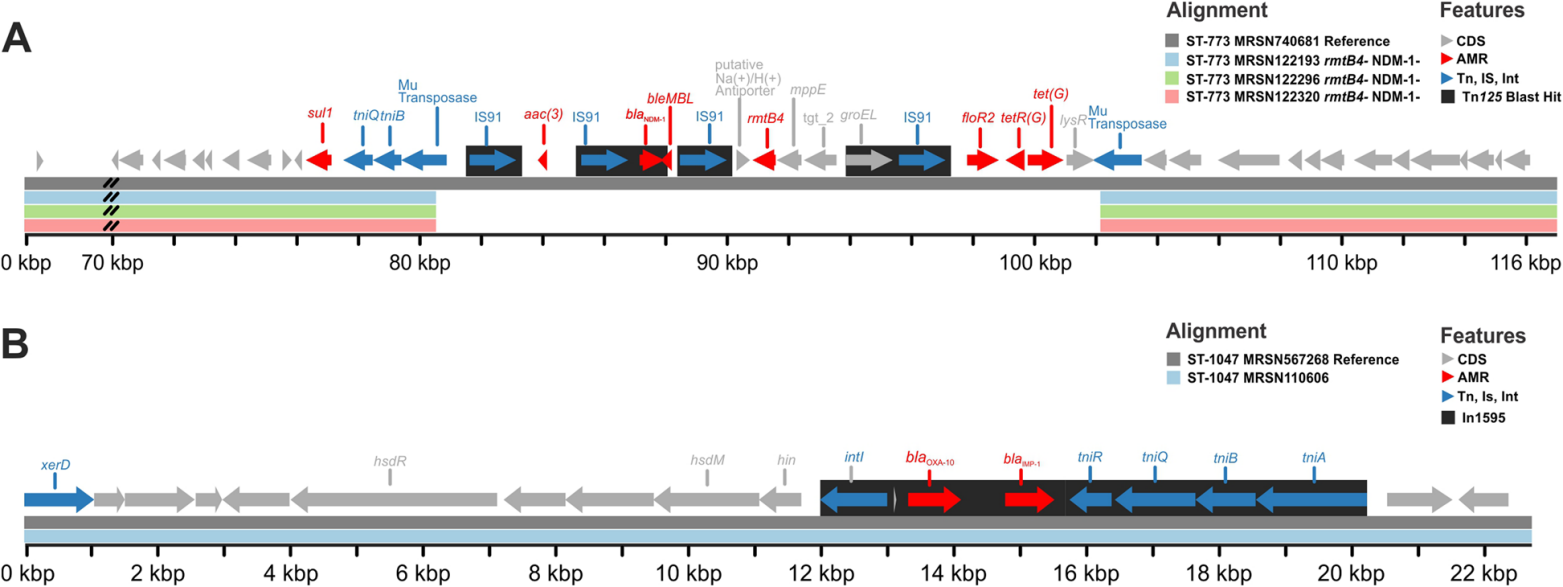
Genetic context of major resistance genes in *P. aeruginosa* epidemic clones. **A** Chromosomal co-localization of *rmtB4* and *bla*_NDM-1_ in ST-773 isolates. Reference is a 117 kb chromosomal fragment from a complete assembly of MRSN740681. **B** Chromosomal *bla*_IMP-1_ in ST-1047 isolates. Reference is a 22.6 kb chromosomal fragment from a complete assembly of MRSN567204

Twenty-four of the 27 isolates carried *rmtB4* and *bla*_NDM-1_. Long-read sequencing detected no plasmids and both genes were chromosomally located within a 117 kb (in reference MRSN740681) integrated conjugative element (ICE) similar to those detected in ST-773 from South Korea, the USA, and Spain [10]. Three isolates lacked both genes because of a 22.4 kb internal region (with homologies to Tn*125*) excising from the ICE (Fig. 6A). As expected, the antibiotic susceptibility profile correlates with the presence or absence of these resistance genes, with only those isolates carrying *rmtB4* and *bla*_NDM-1_ displaying non-susceptibility to all aminoglycosides, 3rd and 4th generation cephalosporins, and the carbapenems. Notably, recurring mutations were observed for the quorum-sensing regulator LasR, for which 9 independent mutational events (including 3 with predicted loss of function) were detected. Strains with loss-of-function mutations in this regulator have previously been shown to modulate immune responses and display survival advantages in animal models of infection [48].

### International spread of an IMP-carrying *P. aeruginosa* ST-1047 clone endemic in Ukraine

Twenty-eight isolates from four countries (Ukraine (*n* = 16), Germany (*n* = 7), The Netherlands (*n* = 4), and Denmark (*n* = 1)) were assigned to ST-1047 (Fig. 2). All formed a single cluster of highly related isolates that were separated by 0–16 alleles. Five isolates were cultured pre-invasion: four from Ukraine facilities # 3, 5, and 7 between 2017 and 2019 and one from a patient receiving care at German facility #2 in 2017 (Fig. 5). Pre- and post-invasion isolates were separated by 14–54 SNPs. Notably, both MRSN 567277 and 122,260 from Ukraine facility #7 were separated by just 14 SNPs, despite being cultured 7 years apart (2017 and 2023, respectively).

All ST-1047 isolates had no plasmids and carried a chromosomally integrated *bla*_IMP-1_ within In*1595*, itself harbored on a 22.6 kb chromosomal fragment (Fig. 6B), which was identical to genomic island PAGI-97B [49]. A representative isolate for this clone was non-susceptible to all antibiotics tested, except colistin and cefiderocol. Finally, LasR was also recurringly mutated in ST-1047, with three of the contemporary isolates (MRSN 110606, 122,251, and 126,503) carrying distinct non-synonymous mutations, including a LOF mutation in 122,251.

### Sporadic detection of *P. aeruginosa* ST-244

Just two isolates were assigned to ST-244 and both were cultured from patients in Ukraine: one from facility #6 in 2017 and the other from facility #4 in 2023. Despite being cultured 6 years apart, they differed by just 28 SNPs. Neither carried a carbapenemase and the earlier isolate (MRSN 567329) only carried nine AMR genes (Table S2, [14]). Interestingly, MRSN 122207 from 2023 acquired an additional three genes, including the aminoglycoside-modifying genes *aac(6′)-Ib4* and *aph(3′)-IIb* and the ciprofloxacin-modifying gene *crpP*.

## Discussion

Since the full-scale invasion of Ukraine by the Russian Federation, multiple reports of XDR bacteria infecting or colonizing patients have been published, both within Ukraine [4–6] and among Ukrainian civilians in other European countries [10–13]. As a direct result of the war, the loss of infection control in Ukraine has contributed significantly to the spread of these organisms. However, the original source of these bacteria remains unknown. Here, we leveraged a unique international collection of XDR *A. baumannii* and *P. aeruginosa* cultured from Ukrainian patients both pre- and post-invasion to explore this relationship. We demonstrate that highly genetically related strains span both time periods and provide the first evidence that strains infecting Ukrainian patients, including Ukrainian refugees in Europe, have been circulating within Ukraine for at least the past 10 years.

The findings in this study reflect previous observations that Gram-negative organisms are the most common cause of war wound infectious complications after hospitalization [50]. This paradigm has been evident since World War II (1939–1945) and has supplanted the previous paradigm from earlier wars, like World War I (1914–1918) and the American Civil War (1861–1865), where infections were predominantly due to Gram-positive and anaerobic organisms [50]. This etiological shift can be attributed to the increased use of antiseptics (and subsequently antibiotics) alongside other surgical advancements throughout the twentieth century, where Gram-positive organisms that were ostensibly introduced to the wound during the initial trauma were eliminated by these methods. In contrast, Gram-negative organisms were often associated with infectious complications later in the healing process [51], suggesting a likely nosocomial origin. This observation has persisted throughout the modern era, with Gram-negative organisms, especially *A. baumannii* and *P. aeruginosa*, being signature pathogens of the Iraq and Afghanistan conflicts, with infections primarily developing following hospitalization [52]. In Ukraine, this same pattern was already observed in soldiers injured in Eastern Ukraine between 2014 and 2107, where initial wound cultures grew predominantly Gram-positive organisms, but these were supplanted by Gram-negative species after 8 weeks of hospitalization [7].

Modern molecular typing has provided greater insights to the global epidemiology of bacteria. For *P. aeruginosa*, a small number of “high-risk” clones are responsible for the majority of infections [53]. In this study, notable high-risk clones belonging to ST-235, 244, 357, and 654 were observed, but ST-773 and ST-1047 were the most prevalent. While ST-773 is increasingly recognized as an emerging global clone [10], reports of ST-1047 have been confined to Asia [54–56], and further surveillance would be required to determine the extent of their global distribution. Both lineages carried acquired carbapenemases, *bla*_NDM-1_ in ST-773 and *bla*_IMP-1_ in ST-1047, making them particularly challenging to treat. In the latter, the *bla*_IMP-1_ gene was chromosomally bound, within a class I integron (In*1595*) itself harbored on a genomic island named PAGI-97B when first identified from an extensively resistant ST-234 strain from Ghana [49]. In both the ST-234 from Ghana and the ST-1047 from Ukraine, PAGI-97B was inserted within the PrrF1/PrrF2 locus. This intergenic locus, which revealed to be a hot spot for chromosomal integration [49], encodes tandem small RNAs involved in the regulation of iron homeostasis and pathogenesis in *P. aeruginosa* [57, 58]. Whether the acquisition of PAGI-97B carrying *bla*_IMP-1_ impacts the virulence of ST-1047 from Ukraine remains to be determined.

*A. baumannii* is also a leading cause of HAIs worldwide and is a prominent cause of infections during conflicts. It was the signature pathogen of war wound infections during the Iraq and Afghanistan conflicts [59] and was frequently isolated from injured Ukrainian soldiers from 2014 to 2017 [8, 60]. A small number of dominant “high-risk” clones are also responsible for most infections worldwide, with two major global clones, known as global clones 1 and 2 (ST-1 and ST-2, respectively), being the most common [61]. However, additional lineages have also been reported in many countries, such as the recent detection of carbapenemase-producing ST-19 in Ukraine and Georgia [62]. In this study, *A. baumannii* was the 3rd most frequently identified organism infecting war wounds, after *P. aeruginosa* and *Klebsiella pneumoniae*, with ST-2 predominating. However, strains belonging to ST-1, ST-19, ST-78, ST-400, and ST-1077 were also represented, and all carried a variety of carbapenemases (Table S1, [14]). Notably, all these lineages have recently been reported in Ukraine and other European countries [4–6, 10–13] and likely represent endemic reservoirs in this region.

Besides the high prevalence of carbapenemase-producing isolates (79%), a remarkable 47% of *A. baumannii* from Ukraine carried an ESBL. These genes have rarely been found in *A. baumannii* globally and high prevalence of ESBL is usually associated with localized outbreaks, with numerous reports from hospitals in the Middle East [63–65]. Here, epidemic clones within lineages ST-400 (GES-11) and ST-78 (CTX-M-115) were responsible for the high ESBL prevalence. Interestingly, besides Ukraine, isolates of ST-78 also carrying the rare combination of a chromosomal *bla*_CTX-M-115_ and a plasmid-bound *bla*_OXA-72_ carbapenemase have previously been reported from around the globe, including from France, Germany, Russia, and the USA [45, 66, 67]. While their origin remains to be proven, it could be hypothesized that these isolates shared a common ancestor with the “Italian clone,” an ST-78 outbreak strain circulating in Italy in the mid-2000s [68], which emerged and spread globally after the acquisition of the *bla*_CTX-M-115_ ESBL gene.

The protracted nature of the outbreak clusters and the low level of genetic divergence accumulated though time (e.g., 14 SNPs between isolates from two patients 7 years apart at the same facility) are suggestive of persisting reservoirs in the hospital, a known capability of *P. aeruginosa* and *A. baumannii* [69]. Here, echoing previous reports [70, 71] and possibly contributing to their persistence [72], we found that both ST-400 and ST-78 *A. baumannii* strains from Ukraine carried a *qacE* disinfectant resistance gene colocalized with the acquired ESBL genes. Further, we found evidence of the similar + *bla*_GES-11_/*qacE*-carrying plasmid, previously described as self-transferable [47], in genetically distinct strains (ST-2 and ST-400) from the same hospital. This is most concerning as recent genomic studies are providing growing evidence of the role of horizontal gene transfer within hospital environments for the maintenance and spread of resistance genes [73, 74]. Altogether, our data suggests that to better understand and control the spread of MDRO, future surveillance efforts will likely need to extend beyond clinical samples in Ukraine and encompass hospital environmental and patient colonization screening surveillance also.

This report has several limitations. First, patient movement is largely unknown, and many patients moved through multiple hospitals over a short period. This, and the lack of environmental sampling discussed in the above paragraph, makes identifying chains of transmission difficult. However, some clear facilities of interest can be identified in the data; for example, nearly identical ST-2 *A. baumannii* are present in Ukrainian facilities #1 and #4 (Fig. 3) and highly related ST-773 *P. aeruginosa* can be identified in Ukrainian facilities #1 and #2 (Fig. 5). Second, albeit a unique resource, a relatively small number of pre-war isolates (54 isolates over 6 years) were available for analysis, constraining the ability to accurately identify potential reservoirs. Finally, recent data associated with Ukraine indicates that *K. pneumoniae* is a leading cause of infections among war wounded [4]. This contrasts with historical reports from Ukraine, where war wound infections due to *K. pneumoniae* were significantly lower than *A. baumannii* and *P. aeruginosa* [7]. For this study, just 3 non-clonal *K. pneumoniae* were available from the pre-invasion period and none was closely related to post-invasion strains.

## Conclusions

The invasion of Ukraine by Russia has placed extraordinary strain on the Ukrainian healthcare system. While this resulted in the proliferation of MDROs, the source of these isolates remains obscure. Herein, we provide the first evidence indicating that many of the strains circulating since 2022 share a close genetic relationship to historical strains from Ukraine. In some cases, direct links to medical facilities within Ukraine can be inferred, but where these reservoirs ultimately exist remains unknown. These data suggest that surveillance efforts should focus on hospital environments with the ultimate aim of identifying and eliminating these sources of infection and enhancing transmission-based infection and prevention control practices.

## Supplementary Information

Additional file 1: Table S1 All isolates in the study. Basic characteristics of all isolates used in this study. Table S2 All clustered isolates in the study. Basic characteristics of all isolates that formed related clusters in this study.

## Data Availability

The genomes of all new isolates have been deposited in the National Center for Biotechnology Information under bioproject ID PRJNA1162747.

## Abbreviations

ESBL: Extended spectrum β-lactamase
HAI: Hospital acquired infection
ICE: Integrated conjugative element
kb: Kilobase
MDR: Multi-drug resistant
MDRO: Multi-drug resistant organism
MLST: Multi-locus sequence type
MRSN: Multidrug-Resistant Organism Repository and Surveillance Network
SNP: Single nucleotide polymorphism
ST: Sequence type
Tn: Transposon
XDR: Extensively-drug resistant
